# On-Farm Composting of Agricultural Waste Materials for Sustainable Agriculture in Pakistan

**DOI:** 10.1155/2022/5831832

**Published:** 2022-08-08

**Authors:** Sarfraz Hashim, Muhammad Waqas, Ramesh P. Rudra, Alamgir Akhtar Khan, Asif Ali Mirani, Tariq Sultan, Farrukh Ehsan, Muhammad Abid, Muhammad Saifullah

**Affiliations:** ^1^Department of Agricultural Engineering, Muhammad Nawaz Shareef University of Agriculture, Multan 66000, Pakistan; ^2^School of Engineering, University of Guelph, Guelph, Canada; ^3^Director Agri. Mechanization, Agricultural Engineering Division, Pakistan Agricultural Research Council, Islamabad, Pakistan

## Abstract

Agriculture is the economic backbone of Pakistan. 67% of country's population resides in rural areas and primarily depends on agriculture. Pakistan's soils are poor in OM and have a low C : N ratio, and the overall fertility status is insufficient to support increased crop yields. Compost is an excellent alternative solution for improving soil OM content. However, this excellent alternative supply in Pakistan has yet to be used. Mass volumes of leaves, grass clippings, plant stalks, vines, weeds, twigs, and branches are burned daily. In this study, different compost piles (P1, P2, and P3) of compost were made using different agricultural and animal waste combinations to assess temperature, pH, and NPK. Results revealed that P3 demonstrated the most successful composting procedure. The temperature and pH levels throughout the composting process were determined in a specified range of 42–45^o^C and 6.1–8.3, respectively. Total nitrogen content ranged from 81.5 to 2175 ppm in farm compost. Total phosphorus concentrations range from 1.33 to 13.98 ppm, and potassium levels, on the other hand, range from 91.53 to 640 ppm in farm compost. The overall nitrogen concentration grew progressively between each pile at the end of a week. The varied concentrations revealed that adding various forms of agricultural waste would result in a variation in the quantity of NPK owing to microbial activity. On-farm composting has emerged as an effective technique for the sustainability of agricultural activities, capable of resolving crucial problems like crop residues and livestock waste disposal. Based on this study's results, the pile (P3) combination shows the best NPK value performance and is recommended for agricultural uses to overcome the OM deficiency.

## 1. Introduction

Waste management (WM) is the topmost environmental challenge across Pakistan. A large share of waste is biodegradable material (organic). Composting is a viable and economical way to utilize waste as a valuable product [1]. Due to the deficiency of organic matter (OM) in Pakistan's soils, the overall fertility status is insufficient to increase crop yield [2]. High temperature, low precipitation, and the clearance of practically all crop residues, except the roots, contribute to low OM. The automated harvesting or threshing of crops, particularly rice and wheat, has exacerbated the problem because rice and wheat straw are primarily burned. For the high productivity of crops, the OM content must be increased. However, since the introduction of chemical fertilizers, conventional sources of OM such as farmyard manure (FYM) and green manure have nearly disappeared. Consequently, the OM content of Pakistan's soils has already hit its lowest point. Compost is an excellent alternative supplement for boosting the soil organic carbon of soil in developed nations. However, this excellent alternative supply has not yet been tapped in Pakistan. Enormous quantities of leaves, grass clippings, plant stalks, vines, weeds, branches, and twigs are burned daily. Suppose this material is decomposed on the farm and incorporated into the soil. Soil fertility can be enhanced, and crop yields greatly increase [3]. Applying FYM, chicken manure, or green manure with N, P_2_O_5_, and K_2_O at specific prices of 150, 100, and 50 kg·ha-1 greatly boosted paddy yield and subsequent wheat yields. Even after the harvest of two crops, the N, P, and K levels of soil were substantially greater than inorganic fertilizers alone [4]. The three primary ingredients for composting are browns, greens, and water. Browns include things like fallen leaves, branches, and twigs. Water, greens, and browns, such as grass clippings, vegetable waste, fruit scraps, and coffee grounds, are required for compost development [5]. Composting feedstock from farms and neighboring sources is common among certain farmers who want to generate a high-quality product at a low cost. If you are worried about the total input cost of your crops, it is always a good idea to compare them to their final market worth [6]. Compost piles, particularly those kept in windrows, can be aerated and mixed using different equipment. Most of these devices utilize a spinning drum equipped with flails for mixing and turning the compost. Double augers are also used in machines with only one elevating conveyor, which elevates and re-deposits the material on an elevating conveyor. On the other hand, these previous art devices cannot be positive in all materials. The material within the compost must be positively exposed to elevated temperatures to cause pathogen death, but it must be entirely inverted. Compost must be stirred and aerated before being used to its best potential by naturally occurring microbes. Because germs require oxygen to function, a pathogen-free end product cannot be produced unless all ingredients are well mixed, aerated, and periodically inverted [7]. The key to successful composting is to control the aerobic decomposition by monitoring the pile or windrow's oxygen, moisture, and C : N ratio. Although yard waste compost is low in plant-available nitrogen, this organic form of nitrogen will be slowly released if applied at high rates. It does, however, contain some phosphorus, potash, calcium, and magnesium levels. Phosphorus (P) and potassium (K) are too low and variable for yard waste compost to be considered a fertilizer [8]. In compost's nitrogen (N) levels, micronutrients like iron are also present in compost. It is possible to speed up the composting process by adding additional N sources such as grass clippings or manure [9].

Management of crop residue is a global ultimatum now-a-day. The amount of agricultural waste produced worldwide is extensive [10]. Also, pollution related to waste disposal techniques demands investigation of environment-favorable methods of dealing with agricultural wastes—the increment of agricultural waste increases aesthetic, health, and environmental concerns. Therefore, secure disposal techniques need to be investigated. Composting has emerged as an environmentally efficient, cost-effective, and safe treatment technology and a productive solution to intensify and sustain agriculture production [11]. Biodegradable wastes, that is, wood shavings, dry fallen leaves, pine needles, sawdust, and coir pith, are mingled to continue adequate and long-lasting humus [12]. Similarly, using appropriate methodology and quality control methods, useful compost substrates can be produced from the different crop residues (Table 1).

There was a need to overcome the issue of low productivity and low C : N ratio. Therefore, this study was designed to provide an option for local farmers to increase their agricultural soils' C : N ratio to meet the country's food requirements. The main objectives of the current research were to select a suitable combination of agricultural waste material to prepare the compost to enhance the soil health and increase the C : N ratio of Pakistan's soil. This investigation aims to enlighten the utmost prominent aspects of the composting progression through its stages, developing the variety of composts, and assisting farmers, researchers, and scientists in selecting a suitable treatment plan for altering the organic waste into a value-added product.

## 2. Methodology

**Figure 1:**
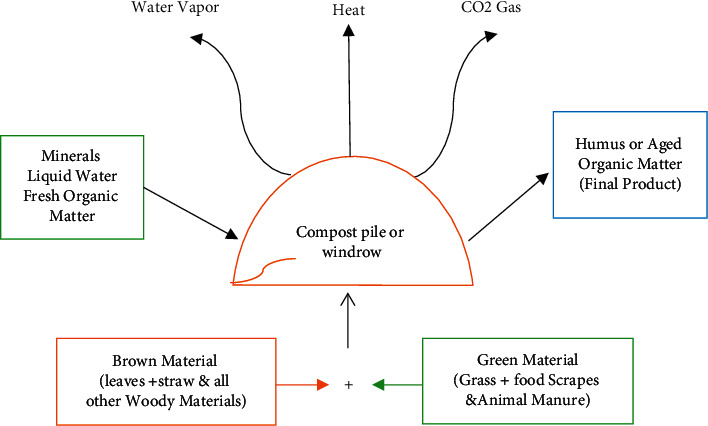
Composting process adapted at SVM's site of MNSUAM.

Composting is a microorganism's controlled breakdown of OM in an aerobic environment. Composting involves using oxygen (O_2_) by bacteria, while they eat OM (Figure 1). It is important to note that active composting creates heat and releases much CO_2_ and water vapor into the atmosphere. The final product's volume and mass are reduced due to the CO_2_ and water losses, which can be half the load of the original components.

We arranged a place for composting at the solid waste management (SWM) site of the Muhammad Nawaz Shareef University of Agriculture, Multan (MNSUAM). The essential requirement for compost generation was to collect the nitrogen and carbon-rich OM. Agricultural waste materials (AWMs) like cotton stalk, rice/wheat straw, maize stalk, fresh grass clippings, and tree prune with leaves were collected from the farms of MNSUAM. After that, these AWMs were stored on the SWM site and mechanically shredded into small chips. Then, we bought animal manure, which is a good source of the vegetation nutrients nitrogen (N), phosphorus (P), and potassium (K). Furthermore, manure yields OM and other nutrients to the soil, such as calcium, magnesium, and sulfur, improving soil fertility and quality.

### 2.1. Compost Piles Making Process

On a half-acre of land, compost piles were formed. Composting may be done in various ways. The most common are static piles, windrows (extended piles), and container composting. It is normal for farmers to use windrows, or large mounds of material rotated over multiple times, for their composting [17]. Therefore, our team selected the windrow or static piles system for our research. The detailed composition of agricultural waste material used in making farm compost is mentioned in Table 2.

Three piles, P1, P2, and P3, were composed of the AWMs mentioned above. The first pile (P1) consisted of one layer of cotton stalk shredded material and then one layer of animal manure, and the second pile (P2) was made with maize stalk shredded material and one layer of animal manure. The third pile (P3) was made with one layer of rice and wheat straw and one layer of animal waste. These piles are 6 feet high, and the width is 8 feet. The turning equipment determines the windrow size, form, and spacing. All the piles were made manually by the layering method, and the length of each pile was up to 20 feet. Natural or passive airflow aerates the piles. The rate of air exchange relies on the pile's porosity. Turning the compost piles improves passive aeration and mixes the components. A light, fluffy leaf windrow can be significantly greater than a moist thick manure windrow. If the pile is too big, anaerobic zones form around the center, releasing smells when rotated. Therefore, small piles up to 20 feet were made, which helped lose heat fast and may not reach temperatures high enough to destroy viruses and weed seeds. After 10–14 weeks, the farm compost was produced at the mass level through a static windrow or pile method.

Compost piles were aerated manually. Static pile systems must be aerated to maintain microbiological activity and appropriate temperatures to satisfy the criteria [23]. Every 3 to 5 days, compost piles were rotated and monitored by compost industry norms [24]. Readings were collected from five locations in each pile and aggregated to ensure that the required standards were met: pH between 5.5 and 9.0, moisture content between 40 and 65%, and temperatures above 62°C for at least three days. For controlling the pathogens during the composting process, the lowest temperature of 62°C has been determined as optimal [25, 26]. After the active compost phase, piles were cured for four weeks to conclude the composting process [27, 28].

**Figure 2:**
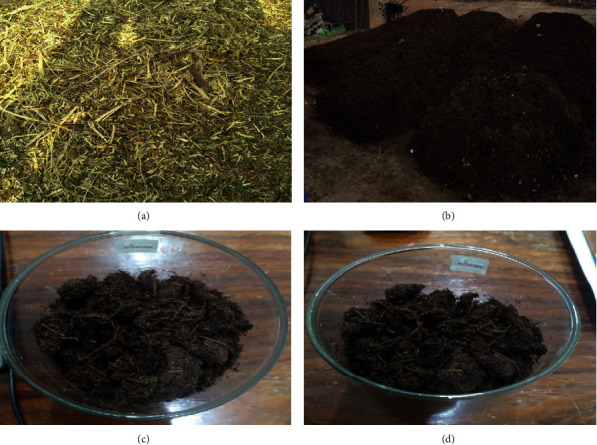
(a) Complete process of compost making from AWMs shredding to final output.

Additionally, for the health and activity of beneficial bacteria, a well-balanced composition of materials is essential. They get their energy and sustenance from carbonaceous brown materials. Protein is synthesized from the nitrogenous green components. Compost piles with an unbalanced composition of brown and green materials have a terrible odor and take a long time to break down. Producing a product full of microbes hungry for nitrogen can also be produced by using too much dry, brown material. Nitrogen from the soil will compete with the plants you want to nourish. The complete process of piles and compost is shown in Figure 2(a) mechanically shredded agricultural material, (b) windrows or piles of compost feedstock (c), and (d) farm compost produced through a static pile or windrow method.

### 2.2. Data Collection

For each test, samples were collected during the composting process every 2 weeks until the 14th week from 2 feet depths in each compost pile. Each sample was 300 grams; 3 samples were taken from piles P1, P2, and P3. The physicochemical properties of each pile were monitored by collecting three samples from the core after 2, 4, 6, 8, 10, 12, and 14 weeks of the process. The samples were analyzed based on the following characteristics: pH, moisture content, OM content, total nitrogen, total carbon, potassium, C : N ratio, OM, temperature, particle size, and curing time [29, 30].

### 2.3. Analytical Observations

The temperatures in each pile were measured using Certeza FT 707 digital thermometers. The evaluation of all parameters was done in duplicate. Water extract (1 : 10) for pH estimation was collected by shaking homogenized samples in distilled water for 15 minutes. A pH meter (HQ440 d) multi-HACH fitted with a glass electrode was used for pH measurement. To determine total nitrogen, samples were wet-digested using a HACH-Digesdahl apparatus, and the digestate aliquot was steam distilled with 40% NaOH. The moisture content of collected samples was determined by the AOAC method [31]. The compost's total phosphorus, potassium, and carbon content were determined by AOAC atomic absorption spectrometer (2000). OM was determined by dry combustion at 550°C. The size distribution of particles in the compost was assessed by sieving the compost in a water-jet sieving system, in which the water pressure of the jet pushed a spraying arm with 34 nozzles to revolve over each sieve [32]. The curing composting stage is frequently followed by a curing period. The materials will continue to decompose slowly during the curing phase. Materials continue to degrade until the remaining microbes eat the last easily degraded source materials. The compost has become relatively stable and manageable [33]. The curing stage of compost usually lasts 3 to 4 weeks.

### 2.4. Statistical Analysis

The physical-chemical analytical results of compost samples obtained from all piles (pH, TN, TP, TK, and OM) are presented as means (*n* = 3) ± standard deviation for all examined piles. One-way ANOVA was used to examine group differences. The difference of *p* < 0.05 was considered statistically significant.

## 3. Results

On the farm, compost was planned to be manufactured on the SWM site with those mentioned above raw agricultural waste such as farm compost, compost from straws, leaves, kitchen, and mud compost. The detailed recipes and results are discussed below. Firstly, manufactured compost is given below.

### 3.1. N-P-K Analysis of Farm Compost

#### 3.1.1. Total Nitrogen Concentration

**Figure 3:**
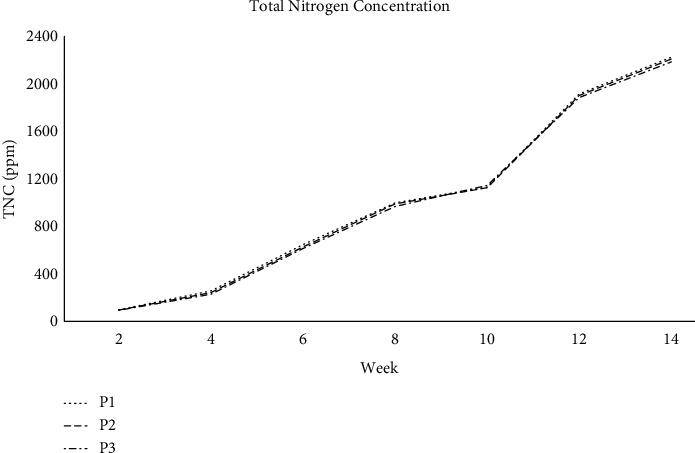
Total nitrogen concentration of farm compost.

Total nitrogen rose somewhat in all reactors during the first four weeks and increased until Week 14 in all windrows. Total nitrogen began to grow due to dry mass loss due to carbon dioxide emissions, water loss due to evaporation, and nitrogen-fixing bacteria activity. The increased total nitrogen content after 20 days of composting showed that it might have been caused by a net loss of dry mass in the form of carbon dioxide and water loss through evaporation induced by heat generated during organic carbon oxidation. From Figure 3, the graph demonstrates that the overall nitrogen content progressively increases week by week, peaking at Week 8 for all piles. The largest concentrations were seen during the last week of composting for all piles, suggesting that the total nitrogen-fixing bacteria may have contributed to the rise in total nitrogen during the later stages of composting. The variance analysis showed significant variations in total nitrogen content between P1 and P2 (*p* < 0.001), and between P1 and P2 (*p* < 0.05). Total nitrogen content did not differ significantly among P1 and SP2 (*p* > 0.05). Total nitrogen loss at the start of composting may be due to ammonia loss due to evaporation at high temperatures.

#### 3.1.2. Total Phosphorus Concentration in Farm Compost

**Figure 4:**
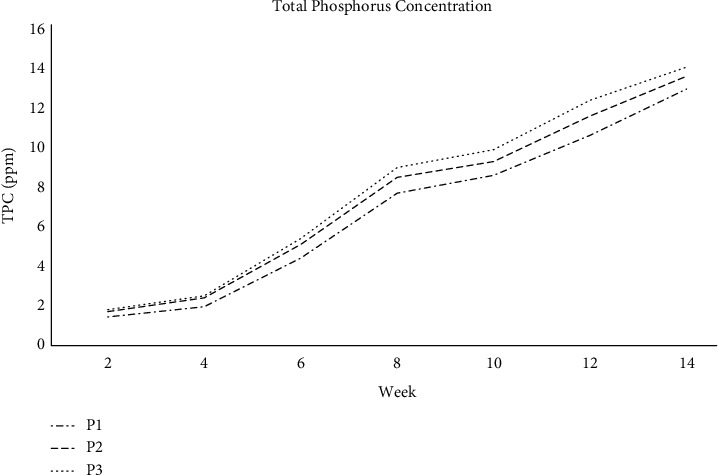
Total phosphorus concentration in farm compost.

The pile's overall phosphorus concentration increases slightly week after week. As seen in Figure 4, the total phosphorus content in all three piles, P1, P2, and P3, steadily rose during the composting process. The maximum concentration was seen in the last week, while the lowest was observed in the initial phases. The concentrations ranged from 1.65 to 13.98 parts per million. The rise in total phosphorus during composting may have been caused by a concentration effect produced by the increased rate of carbon loss associated with the decomposition of organic materials.

#### 3.1.3. Total Potassium Concentration

**Figure 5:**
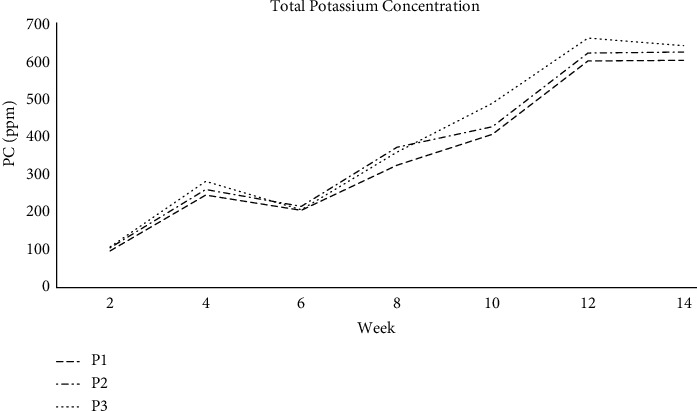
Total potassium concentration in farm compost.

The potassium level is raised weekly in this study but is inconsistent. The graph line in Figure 5 indicates that the potassium value is not growing continuously due to the disruption of microbes in all three piles of farm compost. Weekly levels fluctuate and may be unstable due to the activities of microorganisms found in compost that also require nutrients. Thus, the movement of microorganisms, which require nutrients at specific periods, may result in unstable compost concentration declining before maturity. Most of all, compost concentrations are increasing every week.

### 3.2. C : N Ratio

**Figure 6:**
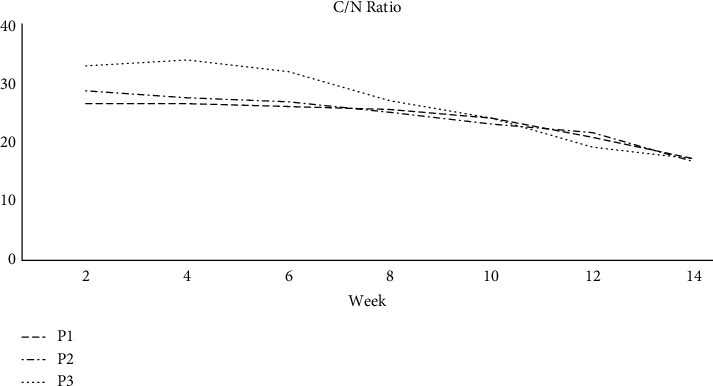
Changes in C/N ratio in P1, P2, and P3 during composting.

Figure 6 depicted a steady drop in the C/N ratio of all substrates during composting because of a greater rate of C breakdown and decreased N losses. P1, P2, and P3 had C/N ratios of 26.5, 28.70, and 33, respectively. The C/N ratio declined in P1 and P2 piles during the observation period. During the first two days of the P3, the C/N ratio climbed to 34.50 and decreased precipitously to 27.0 in the third week. The subsequent decline closely mirrored the trend observed in P1 and P3. After 8 weeks of observation, the C/N ratio in all piles decreased to about 20. After the evaluation period, the C/N ratios of P1, P2, and P3 approached 17.0, 15.50, and 17.0, indicating that the composted material had matured well. According to the C/N ratio revealed by our research, P1, P2, and P3 piles have reached maturity. Variance C/N ratio in P1, P2, and P3 piles substrates with a C/N ratio in the piles P1, P2, and P3 in general. Composting piles containing animal manure have a low C : N ratio in general. Our results and variance analysis revealed significant differences in total nitrogen content among P1 and P2 (*p* < 0.001) and P2 and P3 (*p* < 0.05).

### 3.3. Organic Matter Degradation

**Figure 7:**
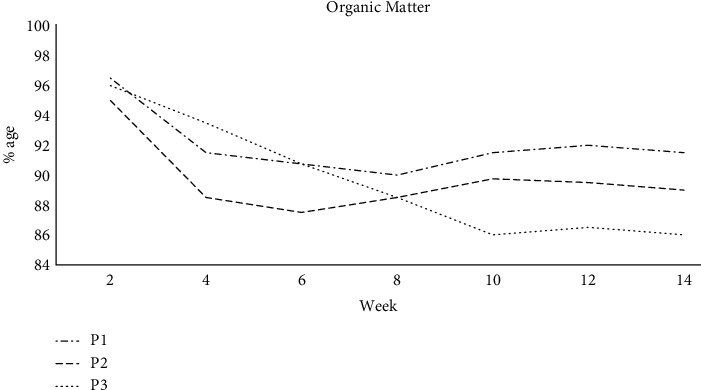
OM degradation in P1, P2, and P3 during composting in 14 weeks.

An analysis of variance revealed the following significant differences in OM loss among the substrates: P1 and P2 (*p* < 0.05), P1 and P3 (*p* < 0.001), and P1 and P3 (*p* < 0.01). Initial OM concentrations in piles P1, P2, and P3 were 96.5%, 95.0%, and 96%, respectively (Figure 7). In most cases, the progressive decline in OM was due to reduced accessible carbon sources. There was just a 3.5% variation between the first and final materials. Lower OM breakdown in C correlated with a low pH and high moisture content, which led to anaerobic conditions and a slower decomposition rate. During weeks 2–4, there was a considerable fall in the P1's OM, which plummeted to 91.50 percent. Until the end of observation, there were no noteworthy changes to the OM in this pile. Due to the relatively quick degradation of organic waste by microbes, P2 decreased the most. The OM content of this pile declined from 95 percent to 88.50 percent after two weeks and to 87.7% after six weeks. Reductions in OM are an excellent indicator of effective degradation processes. After twelve weeks of composting, the overall OM content of P1, P2, and P3 was 92.0%, 89.50%, and 86.5%, respectively.

### 3.4. The pH of Farm Compost

**Figure 8:**
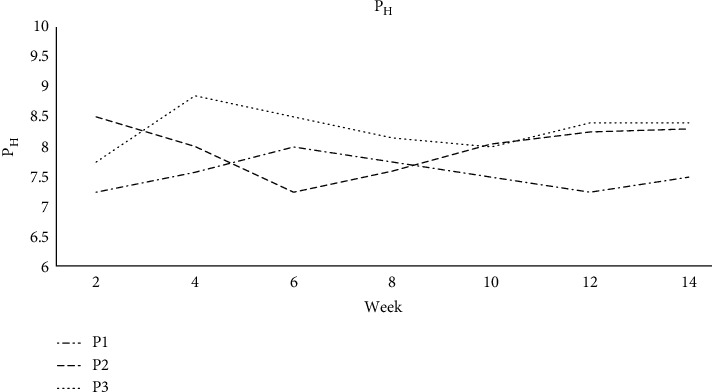
pH in P1, P2, and P3 during composting.

Organic acids are generated during the early phases of degradation. Acidic environments promote fungus growth and lignin and cellulose degradation. Composting neutralizes the organic acids, producing a pH between 7 and 8 for mature compost [34]. In general, the pH decreases during the composting process due to the creation of organic acid and then increases as the acids are consumed [35]. The pH climbed somewhat during the sixth week when the composting process entered its thermophilic phase. It declined slightly to near neutral (7 to 8) when the compost achieved maturity by the eighth week. In the initial composting stage, we observed a fall in pH in piles without lime, which we attributed to the creation of organic acids. As a result of acid generation in aerobic microenvironments, the content of organic acids in composts rises in the presence of low oxygen concentrations.  Figure 8 demonstrates that from Week 1 to Week 2, the pH of substrates P1 and P2 rose from 7.25 to 8.50 and 7.58 to 8.01, respectively. In the P2, the first sudden increase was followed by a drop to 7.75 by Week 1 and then rose to 8.85 by Week 2. While P1 had a lower pH than P3 (*p* > 0.05), there was no significant variation in pH between P1 and P2. There was a substantial difference in pH (p 0.001) between piles P2 and P3.

### 3.5. Curing

Once windrows and piles no longer re-heat after being turned, curing commences. Typically, compost cures for three to four weeks. Curing is a crucial but sometimes overlooked aspect of the composting process. The curing process takes place at mesophilic temperatures. If the active composting stage is reduced or poorly managed, the significance of curing will grow [36]. Immature compost may include high quantities of organic acid, a high C : N ratio, and other harmful properties to plants and crops [37].

### 3.6. Temperature

The primary characteristic that regulates microbial activity throughout the composting process is temperature. Each pile's temperature was recorded before the addition of any agricultural waste. The hottest temperatures recorded in this investigation ranged between 42 and 46°C. The increase in temperature during the composting process is caused by the high temperatures created by microorganisms during respiration and the breakdown of OM. The temperature of composted matter affects the pace of several biological processes. It is critical for microorganism succession, defined as a shift in the quantitative and qualitative makeup of the microorganism population [38]. Temperature variations occur in three phases throughout the composting process: mesophilic, thermophilic, and curing (cooling) stages [39].

Microbes remain active in the thermophilic phase until the compost reaches a particular state of breakdown. The temperature will then return to its initial value during the cooling stage. The temperature of each reactor fell during the composting process in commercial and research compost. The composting process begins in the mesophilic phase. After a few weeks, the temperature rises almost to the thermophilic stage. Both phase microorganisms occur when the temperature increases between 40 and 50°C [40]. According to the temperature at Week 14, each pile was on the verge of curing. When the compost temperature reaches the ambient air temperature, the compost matures. Near the compost piles, the ambient temperature ranged from 12.7 to 27.8°C. Due to the quick decomposition of organic waste, the core temperature of the substrates rose during the first week. P2 exhibited a temperature pattern typical of composting: heating, thermophilic, and cooling phases and ranged from 26.3 to 53°C. Moreover, increasing temperature enhanced substrate breakdown to simpler components. P1 and control substrates experienced lower temperatures than P2 throughout the procedure (25.8–38.8°C and 23.5–41.6°C, respectively). Throughout the composting process, the highest temperatures in piles P2, P3, and P1 were 41.6°C, 53°C, and 38.8°C, respectively.

On days 3, 4, and 5, temperatures above 50 degrees Celsius were reported in the P3 pile. It led to the end of the thermophilic phase of composting, as the temperature progressively lowered to 43.3°C. Between 2 and 3 weeks, the temperature in the P3 core indicated a minor increase followed by a trend like the P2 pile's decrease. Three days of temperatures reaching 50°C in P3 were likely insufficient to remove possible microorganisms and ensure the compost's hygienic safety. Similarly, successful pathogen elimination could not be guaranteed for P2 and P1 heaps. During composting, additions of zeolite and lime substantially impacted the temperature evolution of the substrates tested in this study.

### 3.7. Particle Size

The efficiency of an aerobic breakdown accelerates as particle size decreases. However, smaller particles may diminish the efficacy of oxygen movement within a pile or windrow. Typically, optimal composting conditions are produced with particle sizes varying from 1/8 to 2 inches.

## 4. Discussion

Many researchers have explored the impact of additives on the composting process. Their effect will vary depending on their quantity and characteristics, oxygen availability, substrate composition, C/N ratio, pH, moisture content, and other parameters [41]. The temperature significantly influences microbial activity during composting, but other parameters like moisture, C/N, aeration, and pH may also be essential [42]. Conferring to Antil et al. [43], in the composted substrate, the following temperature phases have been identified: (a) latent phase, which correlates with the time required for microorganisms to acclimate and repopulate the composted substrate; (b) growth phase, in which the biologically significant temperature rises to a mesophilic level; (c) thermophilic phase, when the temperature reaches the highest level; and (d) maturation phase, whenever the temperature decreases to mesophilic and finally to ambient. The magnitude of temperature change shows that the composting process is heterogeneous, reflecting variances in active microbe populations. In our investigation, the temperature in the thermophilic stage was less than in other studies, preventing efficient pathogen elimination. This could be attributed to the high initial moisture content of all three substrates. The maximum temperature was recorded inside the P3, reaching over 53°C for a brief period. Temperatures in the P2 were likewise higher and exceeded those from the control. According to Meng et al. [44], the temperature in the piles grew spontaneously during both the mesophilic and thermophilic stages and was sustained above 55°C for 30 days until it declined during the cooling and maturation stages, which involved cow manure and maize straw composting. During the thermophilic phase, waste stabilization and pathogen killing are most effective [45]. Compost biodegradation activities gradually drop metabolically produced heat to the mesophilic range [46]. Composted materials' physicochemical and biological features influence the appropriate moisture content for composting operations. High moisture causes anaerobic conditions and delayed temperature rise [47]. Changes in water content vary depending on the waste to be composted, aeration, and temperature, but should be between 50 and 60 percent. If the compost becomes too moist, O_2_ diffusion is hindered, and anaerobic conditions emerge, which are undesirable due to the loss of N through denitrification, the rate of gas diffusion decreases. The rate of oxygen uptake becomes insufficient to meet the metabolic demands of the microorganisms [48]. The pH evolution in this investigation was comparable to that observed in the previous study by Huang et al. [49]; they evaluated changes in physicochemical properties of swine, cattle, and chicken manure after 70 days of composting without adding bulking agents. The properties of the three substrates studied differed. The pH of cow substrate increased from 7.86 to 8.36 during the thermophilic phase before dropping dramatically to 7.52 after composting. In the control substrate, a parallel development was seen. After Day 3, the pH climbed for ten days in the thermophilic phase from 7.86 to 8.36, then declined to 7.49 by the finish of composting. Due to nitrification, which creates H+, the pH of compost materials tends to fall in the later stages of composting. In our investigation, reduced OM degradation was associated with higher moisture content and lower pH. Because of the increased moisture, anaerobic conditions developed, resulting in poor composting. Organic matter loss reduces pile weight, lowers the C/N ratio, and represents the effectiveness of degradation processes [50]. An appropriate C/N ratio is critical for calculating the rate of breakdown of organic compounds. Manures, in general, do not have the optimal C/N ratio. Composting is slowed by high C/N levels. Composting with a reduced C/N ratio can result in more nitrogen loss as ammonia gas. To provide degradable OM, low C/N ratios can be adjusted by adding a bulking agent [51]. Nt, C/N ratio, biodegradable organic C, particle size, and composting variables such as temperature and aeration all influence the development of nitrogen compounds. Compost can emit nitrogen compounds like atmospheric nitrogen (N_2_) or nitrous oxide (N_2_O) gases due to nitrification and denitrification. The potential impact of N_2_O on climate change is approximately 300 times greater than that of CO_2_. The transfer of urea to ammonia and further volatilization to the atmosphere might lose up to 50% of the N in newly ejected manure [52]. Our findings and analysis of variance revealed substantial variations in nitrogen concentration between P1 and P3 (*p* < 0.001) as well as between P2 and P3 (*p* < 0.05). Although pH is not a critical element in composting, it is essential in reducing N-losses due to volatilization, which can be relatively high at pH > 7.5 [53].

## 5. Conclusion

This investigation revealed that the altered substrates had a more significant temperature and dry matter content and that the pH evolution was positively influenced. There is also a sign that is adding inorganic elements. In this composting investigation, pile P3 demonstrated the most successful composting procedure. By week, the overall nitrogen concentration grew progressively between each pile. On the other hand, the total phosphorus and potassium concentrations grew weekly. The varied concentrations revealed that adding various forms of agricultural waste would result in a variation in the quantity of NPK owing to microbial activity. There is a possibility that the compost employed in this study might be used for agricultural purposes, given the nutrient performance, or NPK, continues to improve week after week. In this study, pile P3 composted the fastest. After a week, the overall nitrogen content increased between each pile. The varying concentrations demonstrated that adding different types of agricultural waste will change the amount of NPK due to microbial activity. The nutrient performance, or NPK, of the compost utilized in this study may be used for agricultural purposes. In conclusion, the compost can be used for agricultural purposes, with pile (P3) exhibiting the best NPK value.

## Figures and Tables

**Table 1:** Treatment methodologies of different types of crop residues for composting.

Sr. No.	Type of waste	Physicochemical characteristics	Methodology	Quality control methods	Final products and uses	Results	References
1	Rice straw	In a solid: distilled water ratio of 1 : 20 (w/v dry weight basis), *EC* and pH were tested in aqueous extracts of rice straw, oilseed rape cake, poultry manure, and compost.	In 90 days, the composts were ready to use.	Composting	Rice straw composted with oilseed rape cake and poultry manure affects the growth and soil properties of the faba bean (*Vicia faba* L.).	1—The feasibility and the benefit of compost without chemical fertilizer demonstrated the feasibility of sustainable agronomic performance of faba bean using locally available recycled organic materials.	[13]
						2—During composting, total organic C concentrations decreased marginally for all mixtures, while compost N enhanced.	

2	Corn waste	The mixture's temperature rose to >40°C within one week of the onset of *CSC* composting. The thermophilic phase (>40°C) temperatures were sustained for the first seven months of the nine-month composting period.	Compost pH was measured in a 1 : 2 slurry of 25 g compost and water.	Composting	Composting has long been used for the management of manure on farms.	1—Composting alternations in biomass, nitrogen, and 813 C and 814 N content.	[14]
						2—Highly recalcitrant composts that can be stored in nonmineral soil fractions for a long time. During composting, the natural abundance tracer technique's sensitivity to identify their soil's fate increases as a more homogeneous C isotope signature.	

3	Rice straw	At the three main sites, the temperature was determined before turning every two days for the first 16 days and every week before the end of composting (top, middle, and bottom). At 105°C, the moisture content was determined.	Over 90 days, two different mixtures were stacked and composted. The first (C1) mixture contained sewage sludge and wheat straw, while the second (C2) contained sewage sludge, wheat straw, and wood sawdust.	Composting	The inclusion of wood sawdust raises the compost's nitrogen content, resulting in a mildly alkaline compost that affects seed germination by lowering sewage sludge's phytotoxicity.	Temperature (in the thermophilic process, >55°C) and moisture content (30%). The required maturity level for pH (6.73 for C1 and 7.19 for C2) and EC (1.81 mS/cm for C1 and 1.32 mS/cm for C2) were met.	[15]

4	Rice straw	1—pH = 7.886	A laboratory-scale bin composter reactor in a cone shape was utilized during the composting process.	Composting	Depending on the temperature findings for composting mixture at an aeration rate of 0.6 L/min·kg, the compost can be used without limit. There are no pathogens or weed seeds left.	The composting mixture's final C : N ratio was 11. All composting varieties' pH and moisture contents were 7–8 and 40–70 percent, respectively.	[16]
		2—Moisture content % = 7.40					
		3—Total carbon % = 41.6					
		4—Total nitrogen % = 2; C : *N* = 20.83					
		5—Phosphorus % = 0.08 and potassium % = 0.34.					

**Table 2:** Physical characteristics of common farm substrates used to compost deadstock.

AWMs	Total N (g/kg)	C/N ratio	pH	Total P (g/kg)	Total K (g/kg)	Reference
Animal manure	22	15	9.4	3.9	23.2	[18]
Rice straw	8.7	47	6.8	1.1	—	[19]
Rice straw	0.64^1^	61.3	7.6	0.21^1^	1.12^1^	[20]
Wheat straw	5.24	73.8	6.93	0.62	19	[21]
Maize straw	9.41	46.5	7.03	0.93	22.93	[21]
Rice straw	8.51	49.1	7.82	0.88	25.31	[21]
Wheat straw biochar	1.38^1^	38	7.03	0.45^1^	1.06^1^	[22]

^1^Values in percentage of g/Kg. Total *N* = total nitrogen, total *P* = total phosphorus, total *k* = total potassium.

## Data Availability

The data used to support the findings of the study can be obtained from the corresponding author upon request.
